# Incidence of Typical Neutrophil Count With Fy(a-b-) Status Among Hematology Referrals for Neutropenia at an Urban Safety-Net Hospital

**DOI:** 10.1155/ah/2488148

**Published:** 2025-09-16

**Authors:** Anya Parekh, Adam Lerner, Reggie R. Thomasson, J. Mark Sloan

**Affiliations:** ^1^Department of Internal Medicine, University of California Davis Comprehensive Cancer Center, Sacramento, California, USA; ^2^Department of Internal Medicine, Boston University Chobanian & Avedisian School of Medicine, Boston, Massachusetts, USA; ^3^Department of Pathology and Laboratory Medicine, Boston University Chobanian & Avedisian School of Medicine, Boston, Massachusetts, USA

**Keywords:** ethics, neutropenia, white blood cell count

## Abstract

**Background:** Duffy-null associated neutrophil count (DANC) causes neutropenia without clinical sequelae. 25%–50% of people of African ancestry in the United States are thought to have Fy(a-b-) status and are often erroneously identified as having pathologically low neutrophil counts.

**Results:** We performed a retrospective chart review of new neutropenia referrals to the Hematology Clinic at Boston Medical Center (BMC) to evaluate diagnostic patterns for Fy(a-b-) status. 103 new referrals for neutropenia were made from 1/2020 to 2/2022, of which 78 were included for further analysis. DANC was the etiology for low neutrophil count in 64.1%, 82% of whom were African American or Black or were born in an African or Caribbean country. 66% of these patients underwent confirmatory blood bank testing, and 97% of patients tested were confirmed to have Fy(a-b-) status. The average cost of a laboratory visit for patients with typical neutrophil count with Fy(a-b-) status was on average lower, but not negligible, than those without ($363.82 vs. $737.93; *p* < 0.005). These patients were also statistically less likely to have a follow-up appointment (*p*=0.039).

**Conclusions:** Expanded use of serological Fy(a,b) antigen testing for patients with chronic, asymptomatic neutropenia could reduce the cost of care and referrals to the hematology clinic.

## 1. Introduction

Neutropenia, defined as a reduction in the absolute neutrophil count (ANC) below the normal range for the age and ethnic origin of the affected subject, and leukopenia account for 6%–27% of referrals to hematology [1–3]. In the United States, 25%–50% of people of African ancestry are thought to have a clinical phenotype known as typical neutrophil count with Fy(a-b-) status, or Duffy-null associated neutrophil count (DANC), and are often erroneously identified as having neutropenia [4, 5]. Fy(a-b-) phenotype is correlated to homozygosity of a single nucleotide polymorphism in the promoter region of ACKR1, which encodes a decoy chemokine receptor, and may protect against malaria infection [6]. Individuals with this phenotype have clinically asymptomatic chronic ANC, with ANC between 500–1500 × 10^3^/μL [7, 8]. While DANC is present in 80%–100% of those of sub-Saharan African ancestry and < 1% of those with European ancestry, categorization of this phenotype as leukopenia, and thus, outside of the “normal” range, pathologizes non-Whiteness and results in unnecessary referrals [9, 10]. Accordingly, the European Hematology Association and the EuNet-INNOCHRON COST Action working group have recommended the term “ACKR1/DARC-associated neutropenia (ADAN)” instead of ethnic neutropenia to reflect a genetic rather than racial basis, while the American Society of Hematology utilizes “Duffy-null associated neutrophil count.” [11].

Referrals for neutropenia evaluation in patients with DANC are common. A 2016 study at Montefiore Medical Center in the Bronx, New York, found that only 4 patients out of 228 referred for neutropenia evaluation, 72% of whom were Black/African American or Hispanic, subsequently developed a clinically significant hematologic diagnosis requiring ongoing care [12]. A 2023 pediatric study in Detroit found that leukopenia/neutropenia referrals accounted for 12.6% of total hematology referrals, and Duffy null phenotype-associated neutrophil count was identified clinically or through Fy(a,b) antigen typing in 64.8% [13].

Unnecessary referrals for DANC have important healthcare utilization implications, especially for uninsured or underinsured patients [14, 15]. For hospital systems, especially those enriched with patients with the Duffy null phenotype, these referrals take up valuable resources and delay evaluations for patients with actual hematologic conditions. Boston Medical Center (BMC) is a tertiary care center that primarily serves low- and middle-income African American, Hispanic, and immigrant communities [16, 17]. We performed a retrospective chart review of new neutropenia referrals to the hematology clinic to evaluate diagnostic patterns for typical neutrophil count with Fy(a-b-) status [18].

## 2. Methods

A retrospective chart review of the electronic medical record was performed for all patients referred to the BMC Hematology Clinic with the primary problem of “neutropenia” from January 2020 to January 2022 after obtaining Institutional Review Board approval. Patients were excluded if they were referred but never evaluated in clinic, had a primary hematologic malignancy, or were seen in hematology clinic at least once in the previous year. Data on patient demographics was collected, including age, gender identity, self-reported ethnicity/race, and language preference. Laboratory data collected at the time of consultation included white blood cell (WBC) count, ANC, hemoglobin, platelet count, and Fy(a,b) phenotyping if performed. WBC and ANC nadirs were also recorded if available. Patients who identified as Black, African American, or were born in a Caribbean or African country were counted as “Black” for the purpose of statistical analysis. In some cases, patients did not identify a race or ethnicity in the Demographics tab of Epic, and the chart was reviewed for country of birth. Hematology consult notes were scrutinized to identify the final diagnosis, number, and type of laboratory testing that was ordered. DANC was considered in patients with a history of low neutrophil counts (usually over years) with no clinical evidence of recurrent infections and no concomitant myelosuppressive medications or relevant medical conditions. Antigen testing for Fy(a,b) was pursued in patients meeting these criteria when the test became available in mid-2021. The average cost of a laboratory visit was calculated for each referral using the Outpatient Price Transparency Tool from the BMC billing department, which is based on chargemaster prices for the hospital. The Students' *t* test and logistic regression modeling were used for statistical analyses.

## 3. Results

Between January 2020 and January 2022, 103 patients were referred to the BMC Hematology Clinic for neutropenia. Of those, 18 referrals were never seen in clinic, and 7 had already been seen or had a pre-existing diagnosis of benign ethnic neutropenia. These patients were excluded, and 78 patients were included for subsequent analysis. Demographic and laboratory data were abstracted from the electronic medical record and summarized in Table 1. 67.9% of patients identified as Black, African American, Cape Verdean, Haitian, or were born in an African country. The remaining 25 patients identified as White (10.2%), Hispanic (12.8%), Vietnamese (3.8%), or did not have any available racial/ethnic data in Epic (“Other; ” 5.1%). Fifty-five percent (55%) of patients were female, and the primary language spoken was English (69.2%). The average WBC was 4.1 × 10^3^/μL (range: 1.8–25.7), and the average ANC was 1.7 × 10^3^/μL (range 0.3–3.9). WBC did not vary by race, gender, age, ANC, platelet count, hemoglobin, or clinical/laboratory determination of Duffy null status in a statistically significant manner by multiple logistic regression analysis.

Table 2 shows final diagnoses for patients whose primary etiology of neutropenia was not attributed to Duffy null status. Commonly ordered laboratory evaluations included complete blood count with differential, peripheral blood smear, immature granulocyte count, comprehensive metabolic panel, B12 and folate levels, HIV1 and HIV2 serologies, HBV and HCV serologies, antinuclear antibodies, lactate dehydrogenase, type and screen, and Fy(a,b) antigen testing. Several patients were also tested for serum or urine electrophoresis, serum immunofixation, copper, or underwent bone marrow biopsy.

In total, 50 patients (64.1%) were found to have DANC as the primary etiology for WBC outside of standard range. Thirty-three of these patients underwent confirmatory Fy(a,b) antigen testing in the blood bank, and 97% (32/33) of patients tested were Fy(a-b-). Patients with Duffy null status were more likely to be Black or African American (*p*=0.002), undergo Fy(a,b) antigen testing (*p*=1.9 × 10^−6^), and have a lower cost of workup (*p*=1.23 × 10^−5^). The average cost of visit for patients with DANC was significantly lower at $363.82, compared to $737.93 (*p* < 0.001). The cost of visit was inversely related to presence of Duffy null phenotype (*p*=2.1 × 10^−5^) and positively related to number of follow up visits (*p*=0.008), but not statistically significantly associated with age, gender, race/ethnicity, WBC count, ANC count, or other laboratory indices.

## 4. Discussion

On par with other studies, 64.1% of referrals for ‘neutropenia' from 2020 to 2022 at our institution were found to have DANC, which was inversely associated with number of follow up visits and total cost of visit. Patients who received a clinical “diagnosis” of DANC often did not undergo any laboratory testing at all, and cost of visit for these patients accordingly is decreased. There was 97% concordance between clinical suspicion of DANC by the consulting hematologist and laboratory confirmation of Fy(a-b-) status.

In our study, patients were primarily referred by primary care providers (PCPs) and occasionally by inpatient providers after neutropenia were detected on inpatient evaluation. Gay et al. have suggested that Fy(a,b) typing by PCPs may have utility as part of a workflow for neutropenia referrals to hematology, with which we concur [13]. Further work must be done to identify ways to prevent unnecessary referrals to the hematology clinic, perhaps by screening Fy(a,b) typing for patients satisfying a clinical phenotype (such as lack of previous infectious history, chronic or lifelong low neutrophil count, and negative hepatitis and HIV serologies). Individuals meeting these criteria could be triaged by e-consult, and in patients whom another cause of neutropenia is considered, in-person assessment and further diagnostic workup could be pursued after screening with Fy(a,b) status.

Additionally, under-recognition of DANC has important health equity implications. Vastola et al. found that 41.4% of prostate cancer clinical trials excluded patients with typical neutrophil counts with Fy(a-b-) status, and lowering ANC cutoff for patients with DANC could increase the number of Black participants [19]. Though DANC does not increase risk of infection, patients with this phenotype experience more frequent dose reductions and interruptions in chemotherapy for neutropenia, putatively impacting survival [20, 21]. Multiple studies, including ours, have shown an increased burden of unnecessary referrals for these patients, incurring significant costs and logistical challenges such as transportation, childcare, and workday interruption. A recent study showed an incremental net monetary benefit of $15 for universal screening for Fy(a,b) status among pediatric cohorts; this study should be repeated in adults [22].

Limitations of this study include the method of data collection for ethnicity and race, which was obtained from the Demographics tab of Epic, and the small sample size for non-Black populations. As described elsewhere, there is no genetic basis to discretely categorize humans [23]. Ethnicity, defined as grouping based on culture or nationality, and race, which is based on skin color, are mutable and self-defined. This nuance could not be captured with our study design. Additionally, Fy(a,b) typing only became available at BMC in mid-2021, and 36% of patients found to have DANC did not have confirmatory Fy(a,b) typing performed. Additionally, patients who were not suspected of having DANC did not undergo Fy(a,b) typing, and so the value of the test as a screening tool was not established. Though DANC reflects normal variation of white cell count, it is a “…label that illustrates how common phenotypes seen in non-White individuals continue to be considered nonstandard in the medical community.” [11] Patients with DANC, which accounted for the bulk of new referrals for neutropenia, represent an unnecessary cost burden to an overtaxed healthcare system. Expanded use of Fy(a,b) antigen testing for patients with chronic, asymptomatic neutropenia could reduce cost of care and referrals to the hematology clinic.

## Figures and Tables

**Table 1:** Demographic and laboratory data.

Variable	Black	White	Hispanic	Vietnamese	Other^b^	Total or average	*p* value
Race and/or ethnicity^a^ (%)	53 (67.9%)	8 (10.2%)	10 (12.8%)	3 (3.8%)	4 (5.1%)	78	—
Female	29	5	5	2	2	43	—
Primary language is English	44	5	3	1	1	54	—
Age (range)	47 (22–80)	44 (22–78)	44 (26–78)	55 (42–75)	46 (27–72)	46 (22–80)	—
Average WBC (range) at consultation	3.9 (1.8–7.4)	6.8 (2.7–25.7)	4 (2.3–8.1)	3.5 (2.7–3.9)	3.4 (2.2–4.9)	4.1 (1.8–25.7)	—
Average ANC (range) at consultation	1.6 (0.7–3.5)	1.7 (1–2.5)	2 (0.9–3.9)	1.9 (1.6–2.1)	3.4 (0.3–2.6)	1.6 (0.3–3.9)	—
Average HGB (range) at consultation	13.5 (10.9–15.9)	13.9 (12.9–15.9)	13.7 (12.0–16.4)	10.7 (8.7–13.3)	13.4 (11.1–15.7)	13.4 (8.7–16.4)	—
Average PLT (range) at consultation	222 (137–380)	193 (144–237)	226 (183–276)	267 (192–328)	209 (165–289)	221 (137–380)	—
Average cost of visit (range)	$474.38 (0–1277)	$581.38 (0–1256)	$525.70 (110–1104)	$435.67 (202–704)	$624.00 (160–1229)	$498.12	—
DANC (%)^e^	41 (77.4%)	1 (12.5%)	5 (50%)	1 (33%)	2 (50%)	50 (64.1%)	*p*=0.002^d^
Average WBC for DANC (range)	3.8 (1.8–5.8)	4.4 (−)	3.6 (3.2–4.3)	2.7 (−)	3.2 (3.0–3.3)	3.8 (1.8–5.8)	*p*=0.259^c^
Average ANC for DANC (range)	1.5 (0.7–3.5)	1.5 (−)	1.8 (1.4–2.8)	1.6 (−)	1.4 (1.3–1.5)	1.6 (0.7–3.5)	*p*=0.226^c^
Average cost of visit for DANC (range)	$361.07 (0–1056)	$202	$375.20 (187–648)	$202	$553.5 (370–737)	$373.82 (0–1056)	*p* < 0.001^d^
Fy(a,b) antigen testing (number Fy(a-b-))	26 (26)	1 (1)	3 (3)	2 (1)	1 (1)	33 (32)	—
Number of follow up visits	10	16	5	0	0	31	—

*Note:* Laboratory values for WBC, ANC, and PLT presented as (cells × 10^3^/μL) and for HGB as g/dL. PLT = platelet, HGB = hemoglobin.

Abbreviations: ANC = absolute neutrophil count, DANC = Duffy-null associated neutrophil count, WBC = white blood count.

^a^Data obtained from Demographics tab in Epic, reflects self-identified race or ethnicity. “Black” includes individuals who selected the following races or ethnicities: Black, African American, Caribbean, Cape Verdean, or African origin.

^b^Individuals who had no race or ethnicity identified in Epic.

^c^By Student's *t*-test assuming unequal variance (heteroscedastic). Due to low *n* for non-Black populations, patients with Duffy null status were compared to non-Duffy null status. Multiple variate logistic regression was also performed with WBC as dependent variable and did not show statistically significant effect of race on WBC.

^d^By multiple logistic regression. Cost of visit was not statistically significantly influenced by race, but by Duffy status.

^e^By clinical phenotype or antigen testing. Percent reported is proportion of patients with DANC/Fy(a-b-) status, either by clinical or laboratory diagnosis, among patients of that race/ethnicity.

**Table 2:** Final diagnoses in patients in whom DANC was not identified and/or not the driver of neutropenia.

Final diagnosis	*n*	Race/ethnicity	Duffy status	WBC (×10^3^/μL)	ANC (×10^3^/μL)	HGB range (g/dL)	PLT range (×10^3^/μL)
Hepatitis B	1	Black	Unknown	3.6	1.8	15.9	232
Autoimmune disorder	3	Unknown (1), Black (2)	Unknown	2.2–7.4	0.3–1.5	11.9–12.6	207–306
Vitamin B12 deficiency	2	Black (1), Hispanic (1)	Unknown	3.2–3.4	0.9–1.4	12–14	230–237
LGL leukemia	2	Non-Hispanic White (1), Black (1)	Unknown	3.5–25.7	1–1.5	13.1–14.4	205–211
MDS^∗^	3	Non-Hispanic White (1), Black (1), Vietnamese (1)	Unknown	3.4–3.9	1.4–2	8.7–13.9	137–282
Felty's syndrome	1	Vietnamese	Duffy null	3.9	2.1	10.1	328
MGUS	1	Non-Hispanic White	Unknown	3.1	1.6	12.9	207
Prostate cancer	1	Black	Unknown	2.5	1.4	11.8	191
Sepsis	5	Unknown (1), Hispanic (2), Black (2)	Unknown	4.1–8.2	2–3.9	11.1–15.1	169–288
Unknown^∗^	7	Non-Hispanic White (3), Hispanic (2), Black (2)	Unknown	2.3–4.9	0.9–3.3	12.6–16.4	144–242
Drug-related^∗^	2	White (1), Black (1)	Unknown	2.5	0.7	13.1	212
Total	28						

*Note:* MDS = myelodysplastic syndrome.

Abbreviations: LGL = large granular lymphocyte, MGUS = monoclonal gammopathy of uncertain significance.

^∗^Three patients, one with a diagnosis of MDS, one with drug-related neutropenia, and one with unknown cause did not have CBC collected at time of evaluation.

## Data Availability

The data that support the findings of this study are available from the corresponding author upon reasonable request.
